# Targeting Metabolic Diseases: The Role of Nutraceuticals in Modulating Oxidative Stress and Inflammation

**DOI:** 10.3390/nu16040507

**Published:** 2024-02-10

**Authors:** Aida Dama, Kleva Shpati, Patricia Daliu, Seyma Dumur, Era Gorica, Antonello Santini

**Affiliations:** 1 Department of Pharmacy, Faculty of Medical Sciences, Albanian University, 1017 Tirana, Albania; a.dama@albanianuniversity.edu.al (A.D.); k.shpati@albanianuniversity.edu.al (K.S.); p.daliu@albanianuniversity.edu.al (P.D.); 2 Department of Medical Biochemistry, Faculty of Medicine, Istanbul Atlas University, 34408 Istanbul, Türkiye; seyma.dumur@atlas.edu.tr; 3 Center for Translational and Experimental Cardiology, University Hospital Zürich and University of Zürich, Wagistrasse 12, Schlieren, 8952 Zurich, Switzerland; 4 Department of Pharmacy, University of Napoli Federico II, Via D. Montesano 49, 80131 Napoli, Italy

**Keywords:** inflammation, oxidative stress, nutraceuticals, polyphenols, metabolic disorders

## Abstract

The escalating prevalence of metabolic and cardiometabolic disorders, often characterized by oxidative stress and chronic inflammation, poses significant health challenges globally. As the traditional therapeutic approaches may sometimes fall short in managing these health conditions, attention is growing toward nutraceuticals worldwide; with compounds being obtained from natural sources with potential therapeutic beneficial effects being shown to potentially support and, in some cases, replace pharmacological treatments, especially for individuals who do not qualify for conventional pharmacological treatments. This review delves into the burgeoning field of nutraceutical-based pharmacological modulation as a promising strategy for attenuating oxidative stress and inflammation in metabolic and cardiometabolic disorders. Drawing from an extensive body of research, the review showcases various nutraceutical agents, such as polyphenols, omega-3 fatty acids, and antioxidants, which exhibit antioxidative and anti-inflammatory properties. All these can be classified as novel nutraceutical-based drugs that are capable of regulating pathways to mitigate oxidative-stress- and inflammation-associated metabolic diseases. By exploring the mechanisms through which nutraceuticals interact with oxidative stress pathways and immune responses, this review highlights their potential to restore redox balance and temper chronic inflammation. Additionally, the challenges and prospects of nutraceutical-based interventions are discussed, encompassing bioavailability enhancement, personalized treatment approaches, and clinical translation. Through a comprehensive analysis of the latest scientific reports, this article underscores the potential of nutraceutical-based pharmacological treatment modulation as a novel avenue to fight oxidative stress and inflammation in the complex landscape of metabolic disorders, particularly accentuating their impact on cardiovascular health.

## 1. Introduction

Metabolic disorders represent a spectrum of conditions that have become a significant burden on global health systems, with diseases such as obesity, type 2 diabetes, dyslipidemia, and hypertension at the forefront [1]. These conditions not only contribute substantially to global morbidity and mortality rates but also have far-reaching socioeconomic consequences, limiting quality of life for millions of people worldwide [2]. Traditional therapeutic strategies often provide inadequate results, as they do not sufficiently address the intricate biological mechanisms underlying these diseases [3]. In addition to medical interventions, it is crucial to emphasize the role of lifestyle changes in managing these conditions. In particular, oxidative stress, a common factor in metabolic disorders, is often exacerbated by overeating or by physical inactivity [4]. Thus, modifications in diet and exercise are not only recommended but are essential in the prevention and management of these diseases. Lifestyle interventions, including balanced diets and regular physical activity, have been shown to significantly reduce the impact of metabolic disorders, addressing both their symptoms and underlying causes [5].

### 1.1. Molecular Pathways in Metabolic Disorders

**Figure 1:**
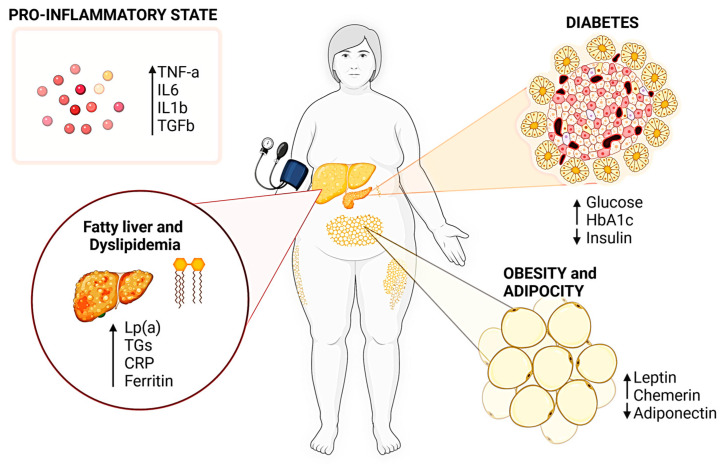
Typical alterations in biomarkers associated with inflammation, adiposity, fatty liver, dyslipidemia, and diabetes. Increased levels of certain cytokines and proteins like IL6, TNF-a, and Lp(a) indicate heightened inflammation and lipid abnormalities, while changes in adipokines reflect altered body fat dynamics. For diabetes, elevated glucose and HbA1c levels along with reduced insulin highlight metabolic dysregulation. Created with BioRender (available at: https://www.biorender.com/ (accessed on 29 January 2023).

The treatment of metabolic disorders requires a nuanced approach, especially considering the key molecular pathways involved. For example, the nuclear factor kappa-light-chain-enhancer of activated B cells (NF-κB) plays a pivotal role in inflammation [6]. It is instrumental in the pathophysiology of atherosclerosis—a primary contributor to cardiovascular disease—by promoting the inflammatory response within arterial walls, leading to plaque buildup and eventual tissue fibrosis [7]. Similarly, the pathogenesis of metabolic and cardiovascular diseases is intimately linked with the activity of proinflammatory cytokines (Figure 1) such as tumor necrosis factor-alpha (TNF-α) and interleukin-6 (IL-6) [8,9]. These cytokines exacerbate metabolic imbalances by promoting insulin resistance, elevating the levels of free fatty acids in the bloodstream, and contributing to a pro-thrombotic state that can precipitate vascular events [10].

To effectively manage metabolic disorders, it is crucial to employ therapies that specifically target and modulate key molecular pathways. Innovative treatments focusing on the inhibition or regulation of NF-κB, TNF-α, and IL-6 hold great promise in addressing the intricacies of these diseases’ healthcare [11]. Such strategic interventions could lead to significantly improved outcomes, particularly for patients at elevated risk of cardiovascular issues stemming from metabolic dysfunctions. This approach represents a more precise and potentially impactful strategy in the complex realm of metabolic healthcare [12].

The pathology of metabolic disorders involves an intricate interplay between oxidative stress and chronic inflammation, which leads to atherosclerosis, tissue fibrosis, and cardiovascular disease [13]. Additionally, they are increasingly prevalent and have emerged as global health challenges posing a serious threat to global health [14,15]. The imbalance of reactive oxygen species (ROSs) and antioxidant defenses is one main factor in the development of these conditions. ROSs and free radicals play a complex role in biological systems, acting as both essential signaling molecules and potential agents of damage [16]. ROSs, a byproduct of normal cellular metabolism, are involved in cell signaling, homeostasis, and defense mechanisms. However, an imbalance in ROS levels can lead to oxidative stress, contributing to cellular damage and a range of metabolic disorders [17,18]. Free radicals, often generated from environmental factors and cellular metabolic processes, can similarly cause oxidative damage when not adequately neutralized by antioxidants. Elevated levels of ROSs can lead to cellular damage. This damage is mediated through lipid peroxidation and protein oxidation, which in turn promote insulin resistance. The impairment of glucose metabolism is often a result of alterations in the phosphatidylinositol 3-kinase (PI3K)/Akt pathway, a critical route for maintaining normal metabolic functions [19]. Additionally, substantial research has identified the key role that inflammatory signaling pathways play in metabolic disorders. Molecular targets such as JNK and IKKβ are pivotal in initiating insulin resistance and endothelial dysfunction. These elements form a critical link between metabolic imbalance and increased cardiovascular disease risk [20,21].

In metabolic disorders, there is a reciprocal relationship between oxidative stress and inflammation: oxidative stress activates inflammatory pathways, which then enhance oxidative stress, creating a vicious cycle [22]. This intricate interplay hastens the progression of metabolic diseases and highlights the critical need for research into comprehensive therapeutic strategies that address these underlying molecular pathways. For example, the modulation of pathways like the Nrf2 signaling pathway, which controls the expression of antioxidant proteins, and the NF-κB pathway, which regulates inflammation, could provide targeted intervention strategies for restoring metabolic and cardiovascular balance [23,24].

### 1.2. Nutraceutical Interventions in Metabolic Disorders

In light of traditional therapies, which have had limited success in treating complex metabolic disorders, nutraceutical interventions have gained prominence as effective alternatives [12]. Polyphenols, resveratrol, and antioxidants like vitamins C and E have been shown to alleviate oxidative stress in metabolic disorders by bolstering the body’s natural antioxidant defenses and reducing the production of reactive oxygen species (ROS) [25,26]. Polyphenols from plants show various biological activities, including the reduction in ROS production by inhibiting enzymes responsible for their generation or binding trace elements involved in free radical formation. Additionally, they scavenge ROS and enhance the body’s antioxidant defenses, targeting enzymes like microsomal monooxygenase, glutathione S-transferase, mitochondrial succinoxidase, and NADH oxidase, which are crucial in ROS generation [27]. Studies have highlighted the antioxidant and anti-inflammatory capabilities of agents like vitamins E and A in mitigating oxidative stress in these disorders [26]. Vitamin E, essential for cell membrane protection, and Vitamin A, crucial for cellular integrity and immunity, are particularly notable. Additionally, polyphenols and omega-3 fatty acids show substantial efficacy in regulating oxidative stress, inflammation, and insulin resistance. This holistic strategy, utilizing diverse bioactive compounds, offers an integrated approach to combat oxidative and inflammatory challenges in metabolic disorders, potentially enhancing the effectiveness of conventional treatments [28].

**Figure 2:**
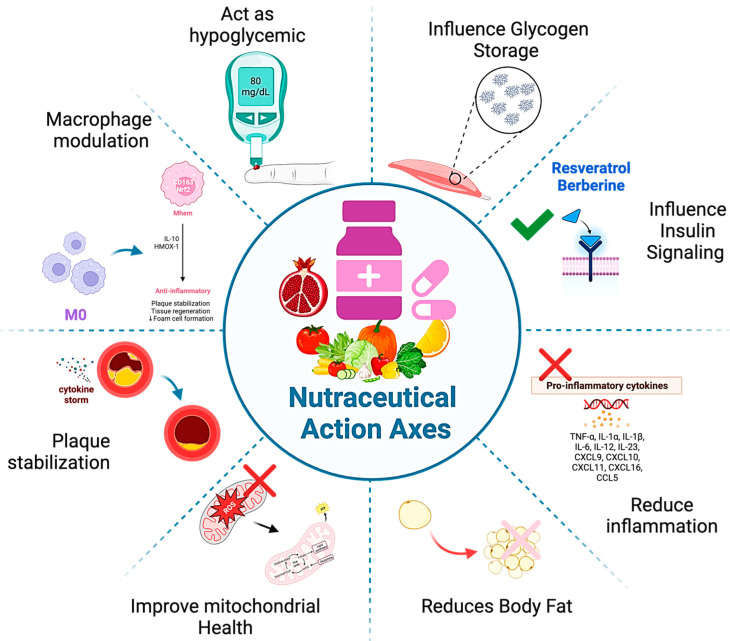
Multifaceted impacts of nutraceuticals: diverse biological pathways through which nutraceuticals exert their effects, encompassing glycemic control, insulin signaling, obesity management, atherosclerosis mitigation, and overall metabolic health enhancement.

In this review, we highlight how nutraceuticals interact with and potentially counteract oxidative stress and inflammation, suggesting that they could rebalance redox states and reduce chronic inflammation. This review emphasizes the significant promise of nutraceuticals in treating metabolic disorders and their extensive effects on cardiovascular health, as supported by the latest research. Supported by recent research, our comprehensive literature search encompassed multiple databases, including PubMed, and Cochrane Library. We employed a broad spectrum of keywords, such as ‘nutraceuticals’, ‘oxidative stress’, ‘inflammation’, ‘metabolic disorders’, and ‘cardiovascular health’, to capture the multifaceted nature of metabolic disorders and the potential impact of nutraceuticals as in Figure 2. Additionally, we strategically combined these terms with specific nutraceuticals and related pharmacological treatments to ensure extensive coverage of the relevant literature.

## 2. Challenges in Metabolic Disorders and Nutraceuticals as a Novel Targeting Strategy

Within the domain of metabolic disorders, a complex interplay unfolds between oxidative stress and inflammation, setting a cascade of events in motion that significantly contribute to the advancement of these pathological conditions. Oxidative stress, marked by an excess of reactive oxygen species (ROS), assumes a central role in initiating and perpetuating this destructive cycle. Beyond their destructive effects on cellular components, ROS functions as signaling molecules that ignite proinflammatory pathways. Recent studies have elucidated the intricate mechanisms through which ROS impair insulin signaling and promote insulin resistance [29,30]. Conversely, inflammation, a hallmark of metabolic disorders, exacerbates oxidative stress through various mechanisms. Recent research highlights how immune cell activation, especially in macrophages and adipocytes, amplifies ROS production by activating NADPH oxidase [31]. Additionally, the recruitment of immune cells to adipose tissue, especially macrophages, plays a pivotal role in shaping the pathophysiological landscape of metabolic disorders. Recent research emphasizes macrophage infiltration into adipose tissue as a critical factor in obesity-related inflammation and metabolic disorders. This infiltration occurs in response to various signals, including chemokines and cytokines, secreted by adipocytes and other immune cells within the adipose tissue microenvironment [32]. This intricate interplay between oxidative stress and inflammation forms a self-sustaining loop, magnifying cellular damage and hastening the progression of metabolic diseases.

New insights are needed in the direction of novel therapeutic strategies to overcome the limitations of traditional approaches, as conventional therapeutic approaches are hindered by their inability to address all the multifaceted roles of oxidative stress and inflammation, as well as the complex signaling pathways involved.

### Nutraceuticals: A Novel and Promising Approach

Nutraceuticals are evolving nowadays as a key player in helping to address these disorders. The bioactive compounds which they are constituted of are derived from natural sources and possess both nutritional value and pharmacological properties [33].

Polyphenols, which are abundantly found in a variety of foods and drinks, including vegetables, fruits, tea, and red wine, are among the most studied nutraceutical agents. Indeed polyphenols—e.g., one of the most studied ones, resveratrol—have obtained considerable attention due to their potent antioxidant effects. A great number of studies have shown that resveratrol enhances endogenous antioxidant defenses and ameliorates inflammation. A recent study showed that this molecule decreased ROS production and reduced inflammation via MAPK pathway inhibition [34]. Furthermore, resveratrol’s ability to modulate the NF-κB pathway and reduce proinflammatory cytokines underscores its anti-inflammatory potential [35]. Polyphenols, derived from plants, exhibit a range of biological activities. Their actions include reducing ROS production by either blocking enzymes responsible for their creation or by binding trace elements that contribute to free radical formation. Furthermore, they actively scavenge ROS and bolster the body’s antioxidant defenses. Specifically, polyphenols target enzymes like microsomal monooxygenase, glutathione S-transferase, mitochondrial succinoxidase, and NADH oxidase, which are key in ROS generation [36].

These compounds offer protection to lipids, shielding them from oxidative harm. The presence of free metal ions amplifies ROS production through the conversion of hydrogen peroxide into the highly reactive hydroxyl radical [37]. Flavonoids, the principal phytochemical compounds in polyphenols, possess a lower redox potential, enabling them to thermodynamically neutralize extremely oxidizing free radicals, including superoxide, peroxyl, alkoxyl, and hydroxyl radicals, through hydrogen atom donation [38,39]. Quercetin, for instance, is renowned for its iron-chelating and iron-stabilizing properties, with trace metals binding at specific locations within flavonoid structures [40]. Additionally, they engage with the aryl hydrocarbon receptor (AhR), a transcription factor that serves as a sensor for organic compounds, initiating the transcription of numerous detoxification genes. These genes encode phase I and II metabolizing enzymes, particularly the cytochrome P450 CYP1 sub-family, Nrf2, and glutathione S-transferase (GST) [41].

Among polyphenols, curcumin has attracted great interest for nutraceutical purposes. It has a wide spectrum of effects, including anti-inflammatory, antioxidant, anticarcinogenic, antimutagenic, anticoagulant, antidiabetic, antibacterial, antiviral, and neuroprotective activities. Curcumin has a potent activity as a scavenger for ROS. It also facilitates the elimination of many reactive oxygen radicals, especially superoxide anions, nitrogen dioxide radicals, and hydroxyl radicals [42]. In addition, the protective effect of curcumin on the cardiovascular system has been widely investigated in recent studies. In these studies, it has been shown that curcumin protects endothelial and vascular functions against damage, and it can activate SIRT1, inhibit the p53/p21 signaling pathway by reducing p53 expression and preventing oxidative stress, and activate NRF2, an important regulator involved in protection against oxidative stress [43,44,45]. Experimental evidence alludes to curcumin’s effectiveness as an antidiabetic agent, primarily through its action on glucose homeostasis. Curcumin activates glycolysis, inhibits hepatic gluconeogenesis, and reduces lipid metabolism, which helps in controlling blood sugar levels. It has shown promising results in diabetic mice models, improving hyperglycemia [46,47]. As an NF-κB inhibitor, curcumin may reduce insulin resistance. Additionally, its role in activating PPARγ contributes to its hypoglycemic effects. Curcumin has also been found to alleviate obesity-related ER stress in tissues, thereby improving insulin resistance and glycemic status, as seen in mouse obesity models. Curcumin effectively reduces systemic inflammation markers like CRP and NF-κB-related cytokines [48,49]. It also lessens hepatocyte damage and oxidative stress while enhancing insulin sensitivity and glycemic control. By activating PPAR-α and PPAR-γ, curcumin supports fatty acid β-oxidation and facilitates weight loss. These actions are crucial in preventing nonalcoholic fatty liver disease, metabolic syndrome, and aiding obesity treatment. Curcumin’s interaction with multiple targets, including lipoprotein lipase, influences the synthesis and breakdown of triglyceride-rich lipoproteins, underscoring its multifaceted therapeutic potential [50].

Hydroxy methyl glutaryl CoA (HMG-CoA) reductase is an enzyme involved in cholesterol biosynthesis in the liver and this enzyme is also target for cholesterol-lowering drugs like statins [51]. It has been shown that curcumin can inhibit liver HMGCoA reductase activity and reduce the activity of the HMGR gene encoding this enzyme in the liver. HMG-CoA reductase inhibitors decrease cholesterol levels by increasing the LDL receptor on the hepatocyte membrane and thus increase the elimination of LDL [52]. Curcumin reduces hepatic cholesterol and total cholesterol levels by suppressing hepatic enzymes HMG-CoA reductase and acyl CoA cholesterol acyltransferase (ACAT). In addition, it inhibits hepatic fatty acid synthase (FAS) activity and increases the beta oxidation of fatty acids. Curcumin specifically downregulated FAS, leading to an effective reduction in fat storage [51,53]. Furthermore, curcumin supplementation was found to suppress the transcription factors PPARγ and CCAAT binding protein α (C/EBPα), which are essential transcription factors in adipogenesis and lipogenesis, mainly in adipose tissue. Curcumin also suppresses the conversion of preadipocytes to adipocytes, which causes the growth and development of adipose tissue. Curcumin exerts this effect in part by suppressing the expression of the transcription factor PPARγ. Therefore, suppression of these transcription factors by curcumin is another potential mechanism by which curcumin contributes to the suppression of adipogenesis [54,55].

Eicosapentaenoic acid (EPA) and docosahexaenoic acid (DHA), specific Omega-3 polyunsaturated fatty acids that are present in fish oil, offer promising potential. Dong et al. [56] found that combining omega-3s with vitamin D3 reduces systemic inflammation, while another study showed anti-neuroinflammatory effects of omega-3 docosapentaenoic acid (DPA) [57]. A meta-analysis of 67 studies involving 310,955 participants highlights the role of PUFAs in lowering the risk of chronic diseases, especially cardiovascular diseases and mortality, with EPA and DHA from marine sources playing a key role [58]. Omega-3 fatty acids impact gene expression, reducing chronic inflammation, a key factor in diseases. They inhibit NF-κB, stimulate PPAR-γ, and regulate G protein-coupled receptors [59]. Understanding these mechanisms is crucial for dietary guidelines and managing inflammation-related diseases. Omega-3s like EPA and DHA influence cellular processes and epigenetic markers, primarily through NF-κB, PPAR-γ, and G protein-coupled receptors [60]. In a study conducted in rats, the effects of EPA and arachidonic acid (AA) on proinflammatory markers were compared. EPA reduced NF-κB activation and MCP-1 secretion, while AGT II and IL-6 levels decreased. Omega-3s inhibit NF-κB, reducing inflammation [61,62]. PPAR-γ interacts with NF-κB, reducing ROS and cytokines [63]. PPAR-γ agonists regulate monocytes and decrease TNF-α, IL-1β, and IL-6 [64]. Studies suggest that the mechanisms of omega-3 fatty acids are not fully understood, and their effects on cardiovascular disease remain uncertain. The optimal dosage and timing for anti-inflammatory responses in humans are unclear [65]. Human diets, influenced by factors like obesity, physical activity, and stress, are complex and may impact epigenetic processes [66]. Omega-3s show promise in managing hypertriglyceridemia and reducing cardiovascular risks [67]. Pharmacological supplements containing DHA + EPA have shown effectiveness in conditions with elevated triglycerides [67,68].

Omega-3 fatty acids’ ability to reduce cytokines and inflammation-related proteins is linked to their influence on gene expression regulation in inflammatory cells. Despite their known benefits, the optimal dosage and duration for these effects are still undetermined, necessitating further research. The American Heart Association suggests that adults consume oily fish, rich in EPA and DHA, at least twice weekly [69]. For those with coronary heart disease, 1 g/day of EPA and DHA is advised, and 2–4 g/day is recommended for hypertriglyceridemia [70]. Supplemental use should be conducted under medical guidance. Understanding the epigenetic impacts of these fatty acids is crucial for developing new dietary guidelines and combating inflammation-related diseases.

Antioxidants have long been a subject of scientific interest due to their potential in mitigating oxidative stress markers, particularly in the context of metabolic disorders. Vitamins C and E, well-known antioxidants, have been the focus of numerous studies examining their role in combatting oxidative stress and associated health conditions [71,72]. One remarkable example of the efficacy of antioxidants is the administration of Silybin complexed with phospholipids, supplemented with vitamins D and E, and milk thistle, as exemplified by the food supplement RealSIL 100D^®^. A comprehensive six-month clinical study involving a cohort of ninety patients with nonalcoholic fatty liver disease (NAFLD) provided compelling evidence of the benefits of the use of this antioxidant-rich combination [73]. In this study, patients with NAFLD, a condition characterized by the accumulation of fat in the liver, were subjected to this novel antioxidant regimen. The results were nothing short of impressive. The antioxidant-rich supplement demonstrated a significant anti-inflammatory effect within the patient group. This effect was manifested through notable improvements in a range of metabolic indicators, including lipid profiles, glucose metabolism, and liver function [74]. The growing acknowledgment of antioxidants in treating endothelial dysfunction and related health issues is notable. Key antioxidants like water-soluble vitamin C (ascorbic acid) and fat-soluble vitamin E are vital in defending endothelial cells [75]. Vitamin C fights damaging free radicals in the cells’ aqueous surroundings, whereas vitamin E shields the cell membranes against oxidative injury [76]. Endothelial dysfunction, often linked to metabolic disorders, is a crucial forerunner to cardiovascular problems [77]. Vulnerable to oxidative stress caused by an imbalance of reactive oxygen species (ROS) and the body’s defenses, these cells can suffer damage and reduced function [78,79]. Therefore, given its clinical significance, the use of antioxidants in treating endothelial dysfunction is showing significant potential.

The remarkable potential of nutraceuticals extends far beyond their actions; it resides in their unique ability to orchestrate a symphony of benefits, harmoniously targeting multiple intricate pathways implicated in oxidative stress and inflammation [80,81,82]. The advent of personalized medicine has introduced a highly promising avenue for optimizing nutraceutical therapy within the realm of these medical conditions [83]. Recent research endeavors have delved into the intricate relationship between genetic factors and individual responses to nutraceutical interventions. This revelation paves the way for the development of tailored nutraceutical regimens precisely aligned with an individual’s genetic predispositions, heralding a significant advancement in the field of personalized healthcare [84].

## 3. Nutraceuticals Usage in Addressing Oxidative Stress and Inflammation in Metabolic Disorders

### 3.1. Historical Perspective of Nutraceuticals

Ancient Ayurvedic medicine, predating Hippocrates’ famous adage “let food be thy medicine and medicine be thy food”, had already recognized the positive influence of dietary and plant consumption on human health [85]. In recent decades, heightened research attention to dietary components has raised public awareness of nutrition. Within this context, the term ‘nutraceuticals’ has emerged, combining the terms ‘nutrient’ (a nourishing food component) and ‘pharmaceutical’ (a drug), implying their potential therapeutic applications, akin to pharmaceuticals. This concept aligns with Stephen DeFelice’s definition of nutraceuticals as “food, or parts of food, providing medical or health benefits, including disease prevention and treatment” [86]. It is worth also noting that the term nutraceutical is being commonly used in the scientific literature and, in some countries, also accepted in marketing products, but has till now no accepted and shared regulatory definition. Consequently, nutraceuticals, when used for animal nutrition, are not subject to specific regulations; Regulation No 1831/2003 should be followed, while compliance with Directive 2001/82/EC is required for nutraceuticals that are used with medical claims or have pharmacological effects. Additionally, when used as ingredients in animal feed, they must conform to Commission Regulation (EU) No 68/2013.

Nutraceuticals are in fact included in the food supplements category. Over recent years, the definition of nutraceuticals has evolved to: “if derived from plant-based foods, nutraceuticals are defined as the phytocomplex, and as the collection of secondary metabolites when originating from animal-based foods, administered in the most appropriate pharmaceutical form”. The key aspect to stress is that nutraceuticals would need studies in vitro and in vivo, e.g., clinical trials, which can assess the appropriate dose, and substantiate their safety, effect, and efficacy against a set health condition, differently from the food supplements which do not require—according to the current regulation—any clinical trials to be put on the market. Alternatively, nutraceuticals may fall under the Foods for Particular Nutritional Uses (PARNUTS) regulatory framework (Directive 89/398/EEC, 1989), encompassing foods for special medical purposes and those designed for specific nutritional requirements. This classification is contingent upon their safety and efficacy, being thoroughly evaluated through in vitro and in vivo studies [87].

### 3.2. Nutraceuticals in Metabolic Syndrome and Cardiometabolic Disorders

Commencing with the influence of metabolic syndrome, a spectrum of health conditions closely linked to dietary habits, there arises a compelling need to establish innovative and sustainable nutraceutical approaches as complements or alternatives to traditional pharmacological treatments, especially for individuals who do not qualify for a conventional pharmaceutical approach. Recent years have witnessed extensive research into the role of nutraceuticals in metabolic diseases, focusing on their oxi-metabolic effects. According to market analysis and the available literature, many nutraceutical products are well-formulated to prevent and manage various health conditions, including diabetes, obesity, and hypertension [88]. This review specifically addresses nutraceutical formulations aimed at improving cardiometabolic disorders and mitigating oxidative stress (Table 1). In this context, Barrios et al. (2017) [89] emphasized the positive impact of a nutraceutical blend comprising red yeast rice, berberine, polycosanol, astaxanthin, and coenzyme Q10. This innovative nutraceutical has demonstrated significant reductions in TC (11–21%) and LDL-C (15–31%) levels, akin to low-dose statins. It also offers a 10% additional improvement in TC and LDL-C for statin-intolerant patients or those not achieving their treatment goals with ezetimibe [89]. Another double-blind crossover study studied a nutraceutical comprising berberine, astaxanthin, policosanol, red yeast rice extract, folic acid, and coenzyme Q10 (namely the commercially available Armolipid Plus) suggested the effectiveness of this nutraceutical in moderate cardiovascular risk situations, especially when a traditional pharmacological approach may not be tolerated well by the patient [90]. A nutraceutical approach using a probiotic Bifidobacterium longum BB536 and red yeast rice extract has been also evaluated. In a recent randomized, double-blind, placebo-controlled trial, a two-week treatment with a nutraceutical blend comprising Bifidobacterium longum BB536 and red yeast rice extract demonstrated significant improvements in the atherogenic lipid profile among individuals with low cardiovascular risk, with high tolerability [91]. Additionally, Tenore et al. aimed to create a novel nutraceutical formulation with gastro-resistant micronized chia seeds and antioxidants, including vitamin E, which was tested in a clinical trial for its impact on human plasma triglyceride levels. This study was conducted in recognition of the well-established benefits of dietary polyunsaturated ω-3 fatty acids on the cardiovascular system [92].

Recently, in a clinical trial, Annunziata et al. demonstrated the Trimethylamine *N*-oxide (TMAO)-reducing effect of grape pomace extract formulated as a nutraceutical rich in polyphenols in particular resveratrol [93]. This study was prompted by the recognition of TMAO as a novel risk factor for cardiovascular diseases (CVDs) and as an oxidative stress biomarker [105]. Polyphenols have a long history as the quintessential antioxidant. They possess potent antioxidant properties, acting as effective scavengers of various oxidants, thanks to the presence of phenolic rings with multiple hydroxyl groups in their chemical structure [106]. In recent years, in addition to the active principles mentioned above, bioactive peptides from plant proteases formulated as nutraceuticals have been considered as the next generation of nutraceuticals [107]. For example, it has been demonstrated that the clinical effectiveness of bioactive peptides with antihypertensive properties hinges on two crucial factors: how well they resist degradation by gastrointestinal peptidases and their ability to be absorbed into the bloodstream [108].

### 3.3. Emerging Nutraceutical Compounds

A very large number of phytochemicals exist, and new compounds will be likely isolated and identified. Recently, there has been growing interest in palmitoylethanolamide (PEA) as a potential nutraceutical due to its natural presence in various plant and animal food sources. Research efforts have focused on understanding the molecular mechanism by which PEA exerts its pharmacological effects. PEA’s binding to PPAR-α initiates heterodimerization with the retinoic acid receptor (RXR), forming an active receptor complex that translocates to the nucleus. This complex binds to peroxisome proliferator response elements, leading to reduced transcription of proinflammatory genes associated with metabolic disorders [109]. In addition, bioactive compounds such as alpha-lipoic acid (ALA) and acetyl-L-carnitine (ALC) have a significant impact on regulating oxidative stress and enhancing mitochondrial function, their primary site of action [110].

### 3.4. Nutraceutical-Based Pharmacological Modulation

Phytochemicals derived from plants and present in nutraceuticals offer valuable tools for the exploration and characterization of diverse receptor types, contributing significantly to our comprehension of their roles in health and disease [111]. While nutraceuticals are not intended to serve as substitutes for pharmaceutical drugs, they play a supportive role in preventive healthcare, especially in addressing conditions frequently associated with metabolic syndrome, including type 2 diabetes, stroke, heart disease, and various cardiovascular issues [112]. Emerging research indicates that dietary phytochemical compounds can influence the endocannabinoid system (ECS), a regulatory system in the body. Compounds like β-caryophyllene (found in edible plants and spices) and 3,3′-diindolylmethane (abundant in Brassicaceae vegetables) act as agonists of CB2 receptors, while falcarinol (present in carrots, parsley, and celery) functions as a CB1 antagonist. Additionally, guineensine (derived from black pepper) and β-amyrin (found in various vegetables) inhibit the re-uptake and enzymatic degradation of endocannabinoids [113]. Activation of CB2 receptors by phytonutrients may help counteract inflammation, while CB1 blockade may have potential benefits for individuals with metabolic syndrome [114].

**Figure 3:**
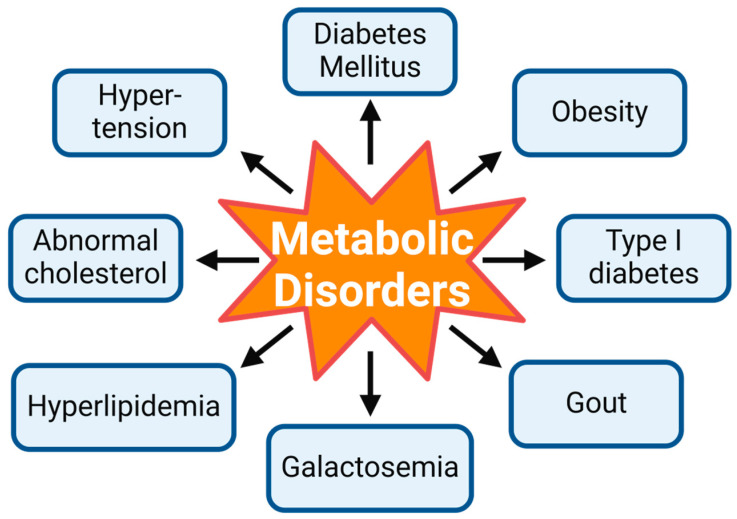
Representative figure of “Metabolic Disorders”, featuring conditions like diabetes type I and II, hyperlipidemia, hypertension, and other diseases.

Moreover, nutraceuticals, as illustrated in Figure 3, exhibit the capability to restore redox balance within the body and modulate the immune response. Oxidative stress, a condition arising from an imbalance between reactive species and endogenous antioxidants, can be alleviated by nutraceuticals due to their possession of antioxidant, antiaging, anticancer, and immunomodulatory properties [115,116]. This multifaceted role of plant-derived compounds in nutraceuticals underscores their significance in promoting overall health and wellbeing.

### 3.5. Potential Usage of Algae-Derived Nutraceuticals

Algae, encompassing a wide range of eukaryotic organisms, extend from unicellular types like Chlorella vulgaris to substantial multicellular varieties, demonstrating their vast diversity [117,118]. These organisms are adept at photosynthesis, thriving in varied aquatic environments, including wastewater. They efficiently convert sunlight, water, and CO2 into valuable bioactive metabolites and oxygen, showcasing their ecological importance [119]. Algae are broadly divided into macroalgae, typically large and found in coastal areas, and microalgae, which are smaller and inhabit both coastal regions and open oceans like phytoplankton. Algae’s role extends to human and animal nutrition [119,120,121]. The reason is that algae are one of the most biologically active resources in nature and contain many bioactive components [122]. Algae are rich in carbohydrates, various amino acids, proteins, fatty acids, and dietary fibers. They also contain polysaccharides, polyphenols, antioxidants, pigments, and other active substances that play important roles in various biological processes such as antioxidant activity, antiviral, antitumor, anticoagulant, and anti-inflammatory responses [121,123]. Due to these numerous immunomodulatory components, they are known to prevent diabetes, oxidative stress, inflammation, and high cholesterol [124]. Because of these potent bioactive molecules, algae are used industrially as nutraceuticals and in a wide range of commercial fields, including pharmaceuticals [121,123].

Polyunsaturated fatty acids, PUFAs, derived from microalgae are important bioactive components with health benefits. Especially polyunsaturated fatty acids such as omega 3 and omega 6 among PUFAs draw a lot of attention. These fatty acids are essential fatty acids and cannot be synthesized in the human body [125]. Algae contain essential fatty acids such as eicosapentaenoic acid (EPA), docosahexaenoic acid (DHA), γ-linoleic acid (GLA), and arachidonic acid (ARA) that have important effects in various metabolic and cardiovascular diseases. For instance, Schizochytrium sp. (dietary marine algae), Crypthecodinium cohnii (dinoflagellate marine algae), Amphidinium sp. (15N enriched dinoflagellates), and Prorocentrum triestinum can synthesize DHA, while Porphyridum cruentum and Chrysophyceae (green algae) can synthesize EPA [126,127]. Arthrospira platensis and Porphyridium purpureum species have been reported as sources of GLA and ARA, respectively [128]. GLA-rich nutraceuticals have also been reported to be effective in the treatment of breast cancer, skin allergies, diabetes, obesity, rheumatoid arthritis, heart disease, high blood pressure, multiple sclerosis, hyperactivity disorder, and neurological problems [129,130].

## 4. Nutraceuticals as Novel Drug Targets

### 4.1. Interaction of Nutraceuticals with Oxidative Stress Pathways

Nutraceuticals can be a tool for support and coadjutant therapy in many health conditions. They aid in regulating oxidative stress, an imbalanced redox state arising from elevated levels of reactive species, and a notably lower presence of endogenous antioxidants in the body. Nutraceuticals, as mentioned, may help to prevent oxidative stress as well as other health conditions, e.g., diabetes, neurodegeneration, organ inflammation, and cardiovascular diseases, which are results of cellular oxidation. Nutraceuticals may be a useful tool to maintain proper homeostasis preventing oxidative stress [131,132] and the onset of good health conditions. Novel approaches in this field are needed, including different pharmaceutical formulations which also include nano nutraceuticals [133], for example, which are better capable of reaching their target and exerting their beneficial health effects. Among them, the prevention and treatment of complicated diseases are notable. These have recently increasingly been the focus of researchers, clinicians, and healthcare providers. What contributes to the popularity of nutraceuticals is their ability to effectively boost the immune system, their widespread availability, affordability, and well-tolerated nature among people [134].

Nutraceuticals contain potent active ingredients that, when administered in controlled doses, offer health benefits without toxicity. This has bolstered consumer confidence, leading to their use in preventing common and chronic ailments such as diabetes [135]. Maintaining the balance between antioxidants and reactive species is vital in preventing diseases, including severe conditions like cardiovascular diseases, neurodegenerative diseases, and renal failure [111]. In more challenging cases such as cancer, pro-oxidant therapy is being explored. Substances like polyphenols and water-soluble vitamin C can induce oxidative stress in cancer cells, disrupting their growth and causing DNA damage [136]. Numerous researchers have explored the use of drug-compound-based nutraceuticals to enhance both their effectiveness and bioavailability in omega-3 polyunsaturated fatty acids, calcium, vitamin D, folic acid, resveratrol, alpha-lipoic acid, zinc, inositol, and probiotics [137]. Nutraceutical formulations with physical and chemical stability entail many challenges. Most phytochemical compounds must be controlled for the negative effects of light, heat, oxygen, elevated humidity, and alkaline pH. Creating a nutraceutical formulation involves understanding the fundamental physicochemical properties of various ingredients, employing appropriate manufacturing techniques, choosing suitable excipients, and incorporating the necessary manufacturing adjustments, as informed by crucial stability studies [138,139,140]. The formulations have an important role in addition to drug interactions in poly-medication treatments. Strict regulation is essential to curb their uncontrolled use and prevent undesirable side effects [88].

### 4.2. Considerations for Clinical Translation and Challenges of Drug Formulations

Clinical translation for nutraceuticals is a current challenge for their potential protective cardiovascular effects due to compounds like resveratrol, cocoa, quercetin, curcumin, glucosinolates (contained, e.g., in *Brassicaceae*), berberine, and *Spirulina platensis* [141,142,143]. A novel lifestyle approach to lower age-associated arterial stiffness represents a clinically significant challenge that could be targeted by identifying nutraceutical approaches to lower CVDs risk. Recently, the role of Apigenin, a flavonoid found in fruits and vegetables, has been studied for its antioxidant, anti-inflammatory, and antibacterial effects [144]. The potential therapeutic effects in the treatment of atherosclerosis, stroke, hypertension, ischemia/reperfusion-induced myocardial injury, diabetic cardiomyopathy, and drug-induced cardiotoxicity have been reported opening the way to explore novel approaches to translational strategies for cardiovascular disease treatment [145]. The challenges that are faced in formulating novel drug targets must be mentioned. They are the focus of many studies. Drug interactions, also known as situations where one active constituent’s activity is influenced by the presence of other constituents, can manifest as food–drug interactions or drug–drug interactions, resulting in potential alterations in the pharmacological response, including alleviation, reduction, or induction of side effects. The latter means (i) various dosage forms, (ii) various formulation challenges, (iii) excipient selection, and many others due to the various steps in the production process [146,147].

## 5. Conclusions

Metabolic disorders, characterized by the complex interplay of oxidative stress and chronic inflammation, pose significant and multifaceted healthcare challenges. Conventional treatment methods—often insufficient in addressing this complexity—call for innovative solutions. Nutraceuticals, along with ongoing research, hold promise for the future in terms of prevention, treatment, and support alongside pharmaceutical therapies. They offer a comprehensive approach to enhancing metabolic health, particularly in cardiovascular wellbeing, by targeting various intricate pathways associated with oxidative stress and inflammation. Emerging nano formulation techniques seek to overcome formulation hurdles, resulting in micronized dietary products and nutraceutical supplements with amplified advantages. Evaluating clinical evidence for each nutraceutical is imperative, as broad generalizations—such as ‘nutraceuticals work’ or ‘nutraceuticals are merely placebos’—lack scientific substantiation. The production process should encompass rigorous monitoring, standardization, valid toxicological studies, precise product characterization, and an understanding of the absorption, distribution, metabolism, and excretion (ADME) characteristics of bioactive components. Realizing the full potential of nutraceuticals for optimizing metabolic health necessitates a blend of robust scientific methodologies and judicious evidence assessment as we navigate this promising frontier.

Nutraceuticals and beyond are the future of many natural substances from vegetal and animal origin in the context of ensuring the optimal productivity of natural resources and sustainability with relevant impact on the circular economy; this is especially the case for countries which possess rich sources of raw materials, where their use must be economically sustainable. It is important to mention once more the importance of novel treatment strategies and proper drug formulation. Almost all naturally occurring compounds like omega-3 fatty acids, flavonoids, and polyphenols like resveratrol are proven to be efficacious. This is due to their strong anti-inflammatory and antioxidant properties, with substantial clinical support for improving cardiovascular and metabolic health. Conversely, there exist nutraceuticals components like curcumin, found in turmeric, which have shown less efficacy, potentially due to a low bioavailability, source quality, and the complex nature of individual metabolic pathways. Therefore, future research directions should include improving bioavailability and personalizing nutraceutical interventions based on genetic and metabolic considerations, aiming to maximize positive therapeutic outcomes.

## Figures and Tables

**Table 1:** Some key nutraceuticals, active compounds, and health benefits.

Nutraceutical	Primary Active Compound	Health Benefits
Omega-3 Fatty Acids [35,58,59]	EPA, DHA	Anti-inflammatory, cardiovascular health
Probiotics [91]	Various live bacteria	Gut health, immune support
Resveratrol [93]	Stilbenoids	Antioxidant, antiaging
Vitamin D [56,57]	Cholecalciferol, Ergocalciferol	Bone health, immune function
Flavonoids [36,94]	Quercetin, Kaempferol, Myricetin	Anti-inflammatory, cardiovascular health, anticarcinogenic, antioxidant
Curcumin [46,49]	Curcuminoids	Anti-inflammatory, antioxidant
Selenium [95]	Selenomethionine, Selenocysteine	Antioxidant, thyroid function, immune health
Coenzyme Q10 [89]	Ubiquinone, Ubiquinol	Antioxidant, heart health
Allicin (Garlic) [96,97,98]	Allicin	Anti-inflammatory, antioxidant
Anthocyanins [99,100]	Cyanidin, Delphinidin	Anti-inflammatory, antioxidant
Soy Isoflavones [101,102]	Genistein, Daidzein	Antioxidant, cardiovascular and bone health, menopausal symptom relief
Lycopene [103,104]	Lycopene	Antioxidant, anti-inflammatory, anticarcinogenic, heart health

## Data Availability

This study did not create new data.
